# FTO inhibition mitigates high-fat diet-induced metabolic disturbances and cognitive decline in SAMP8 mice

**DOI:** 10.1186/s10020-025-01126-4

**Published:** 2025-02-21

**Authors:** Alba Irisarri, Ana Corral, Núria Perez-Salvador, Aina Bellver-Sanchis, Marta Ribalta-Vilella, Roger Bentanachs, Marta Alegret, Juan Carlos Laguna, Emma Barroso, Xavier Palomer, Daniel Ortuño-Sahagún, Manuel Vázquez-Carrera, Mercè Pallàs, Laura Herrero, Christian Griñán-Ferré

**Affiliations:** 1 pHD Program in Biotechnology, Faculty of Pharmacy and Food Sciences, University of Barcelona, Avda. Joan XXIII 27, 08028 Barcelona, Spain; 2 Department of Pharmacology, Toxicology and Therapeutic Chemistry, University of Barcelona, Avda. Joan XXIII 27, 08028 Barcelona, Spain; 3 Institute of Neurosciences of the University of Barcelona, University of Barcelona, 08035 Barcelona, Spain; 4 Department of Biochemistry and Physiology, School of Pharmacy and Food Sciences, University of Barcelona, Avda. Joan XXIII 27, 08028 Barcelona, Spain; 5 Institute of Biomedicine of the University of Barcelona (IBUB), University of Barcelona, Avda. Joan XXIII 27, 08028 Barcelona, Spain; 6 Centro de Investigación Biomédica en Red (CIBER) de Fisiopatología de la Obesidad y Nutrición (CIBEROBN), Instituto de Salud Carlos III, 28029 Madrid, Spain; 7 Spanish Biomedical Research Center in Diabetes and Associated Metabolic Diseases (CIBERDEM) - National Institute of Health Carlos III, 28029 Madrid, Spain; 8 Pediatric Research Institute-Hospital Sant Joan de Déu, 08950 Esplugues de Llobregat, Barcelona Spain; 9 Laboratorio de Neuroinmunología Molecular, Instituto de Investigación de Ciencias Biomédicas (IICB) CUCS, Universidad de Guadalajara, 44340 Guadalajara, Jalisco Mexico; 10 Centro de Investigación en Red, Enfermedades Neurodegenerativas (CIBERNED), Instituto de Salud Carlos III, Madrid, Spain

**Keywords:** m6A, FTO, Epigenetics, Aging, Neurodegenerative disease, Metabolic disorders

## Abstract

This study investigated the effects of fat mass and obesity-associated (FTO) inhibition on cognitive function and metabolic parameters of senescence-accelerated mouse prone 8 (SAMP8) mice fed a high-fat diet (HFD). SAMP8 mice fed an HFD exhibited increased body weight, impaired glucose tolerance, and elevated serum leptin levels. In epididymal white adipose tissue (eWAT), pharmacological treatment with FB23, a well-established FTO inhibitor, increased leptin production and modulated genes involved in lipid metabolism *(Cpt1a, Atgl, Hsl, Fas)*, oxidative stress (OS) (*Bip, Edem*), and inflammation *(Mcp1, Tnfα)*. Expression of hepatic genes related to lipid metabolism *(Cpt1a, Atgl, Mgl, Dgat2, Srebp, Plin2)* and OS *(catalase, Edem)* were modulated by FB23, although hepatic steatosis remained unchanged. Remarkably, FB23 treatment increased m6A RNA methylation in the brain, accompanied by changes in N6-methyladenosine (m6A)-regulatory enzymes and modulation of neuroinflammatory markers *(Il6, Mcp1, iNOS)*. FTO inhibition reduced the activity of matrix metalloproteases *(Mmp2, Mmp9)* and altered IGF1 signaling *(Igf1, Pten)*. Notably, enhanced leptin signaling was observed through increased expression of immediate early genes *(Arc, Fos)* and the transcription factor *Stat3*. Improved synaptic plasticity was evident, as shown by increased levels of neurotrophic factors *(Bdnf, Ngf)* and restored neurite length and spine density. Consistent with these findings, behavioral tests demonstrated that FB23 treatment effectively rescued cognitive impairments in SAMP8 HFD mice. The novel object recognition test (NORT) and object location test (OLT) revealed that treated mice exhibited enhanced short- and long-term memory and spatial memory compared to the HFD control group. Additionally, the open field test showed a reduction in anxiety-like behavior after treatment with FB23. In conclusion, pharmacological FTO inhibition ameliorated HFD-induced metabolic disturbances and cognitive decline in SAMP8 mice. These results suggest that targeting FTO may be a promising therapeutic approach to counteract obesity-induced cognitive impairment and age-related neurodegeneration.

**Supplementary Information:**

The online version contains supplementary material available at 10.1186/s10020-025-01126-4.

## Introduction

Obesity is a global concern whose prevalence is rapidly increasing (Chew et al. 2023). These conditions are closely associated with several comorbidities, including cardiovascular disease, type 2 diabetes, and neurodegenerative diseases. The intricate interplay between genetic predisposition and environmental factors, particularly a high-fat diet (HFD), are major contributors to obesity and associated metabolic disorders (Smith et al. 2023). In recent years, the role of epigenetic changes in regulating metabolism has received considerable attention (Wu et al. 2023; Sibuh et al. 2023). In this context, N6-methyladenosine (m6A) RNA modification has become crucial in various biological processes, including metabolism. Notably, the fat mass and obesity-associated (FTO) gene, which encodes an m6A demethylase, has been strongly associated with obesity and metabolic disorders in genome-wide association studies (GWAS) (Huang et al. 2023a; Azzam et al. 2022; Ran et al. 2020).

FTO is widely expressed in various tissues, including the brain, liver, and adipose tissue. It plays an important role in the regulation of energy homeostasis, body weight, and food intake (Gao et al. 2024). The enzyme catalyzes the demethylation of m6A in RNA, thereby influencing RNA stability, splicing, and translation (Gao et al. 2024). Numerous studies have shown that overexpression of Alpha-ketoglutarate-dependent dioxygenase (FTO) is associated with increased body weight and fat mass. At the same time, its inhibition or deletion leads to a reduction in obesity and an improvement in metabolic parameters (Angelidi et al. 2022; Peng et al. 2019; Liu et al. 2024). Given the critical role of FTO in regulating metabolism, it is an attractive target for therapeutic intervention to combat obesity and related diseases. A number of small molecule inhibitors of FTO have been designed and developed and have shown promise in preclinical studies (Jiang et al. 2024; Prabhakar and Davis 2022; Huang et al. 2023b). Among them, meclofenamic acid (MA)-derived compound FB23 shows improved inhibitory activity against FTO and potential application for further therapeutic approaches (Huang et al. 2019, 2023c; Liu et al. 2021).

New findings indicate a close link between metabolic disorders and cognitive impairment (Amidfar et al. 2024). In humans, epidemiologic data suggests that obesity and HFD are associated with an increased risk of cognitive decline and Alzheimer´s disease (AD) (Lin et al. 2023). Likewise, the association between HFD consumption and AD has been investigated in various mouse models that mimic the major AD pathologies (Choi et al. 2022; Liang et al. 2023). These studies have shown that an HFD can exacerbate Aβ accumulation, neuroinflammation, and cognitive decline in mice. However, the exact mechanisms underlying this relationship in humans remain an active area of research.

A key feature of age-related neurodegenerative diseases is cognitive decline, which is associated with structural and functional changes that affect brain mass and volume and further impair memory (Brito et al. 2023). It is known that m6A methylation is the most common RNA modification in the eukaryotic brain transcriptome, and recent studies have identified m6A as an important regulator of neuronal development and neurotransmission (Mitsuhashi and Nagy 2023; Mathoux et al. 2021). In this context, FTO has also been shown to regulate neurogenesis in adults (Li et al. 2017) and to play a role in memory formation and consolidation (Yen and Chen 2021).

The Senescence-Accelerated Mouse Prone 8 (SAMP8) strain has become a valuable model for the study of age-related diseases, including metabolic disorders and neurodegenerative diseases such as AD (Pačesová et al. 2022). These mice exhibit accelerated aging and develop various age-related phenotypes, making them particularly useful for studying the interplay between aging, metabolism, and cognitive decline. SAMP8 mice fed HFD have been shown to develop obesity (Ding et al. 2024), insulin resistance (Mehla et al. 2014), and cognitive impairment (Mehla et al. 2014; Palomera-Avalos et al. 2017), mimicking the metabolic and neurological changes observed in humans with obesity and metabolic syndrome (Farooqui et al. 2012). This model provides an excellent platform for evaluating potential therapeutic interventions targeting metabolic and cognitive disorders.

However, the FTO inhibition potential therapeutic approach remains to be fully understood in the context of HFD-induced metabolic alterations, particularly in accelerated aging models such as the SAMP8 mice. Oxidative stress (OS) and chronic low-grade inflammation are critical features of obesity and metabolic syndrome (Masenga et al. 2023). These processes contribute to insulin resistance, dysregulation of lipid metabolism, and tissue damage. The interplay of OS, inflammation, and m6A RNA modification in the context of metabolic disorders is essential to understanding the neurodegenerative process in numerous neurological disorders, such as AD (Luo et al. 2021). Therefore, investigating the effects of FTO inhibition on synaptic function in the context of HFD-induced metabolic alterations is crucial for deciphering the intricate relationship between metabolism and cognitive function.

## Material and methods

### Animals

**Fig. 1:**
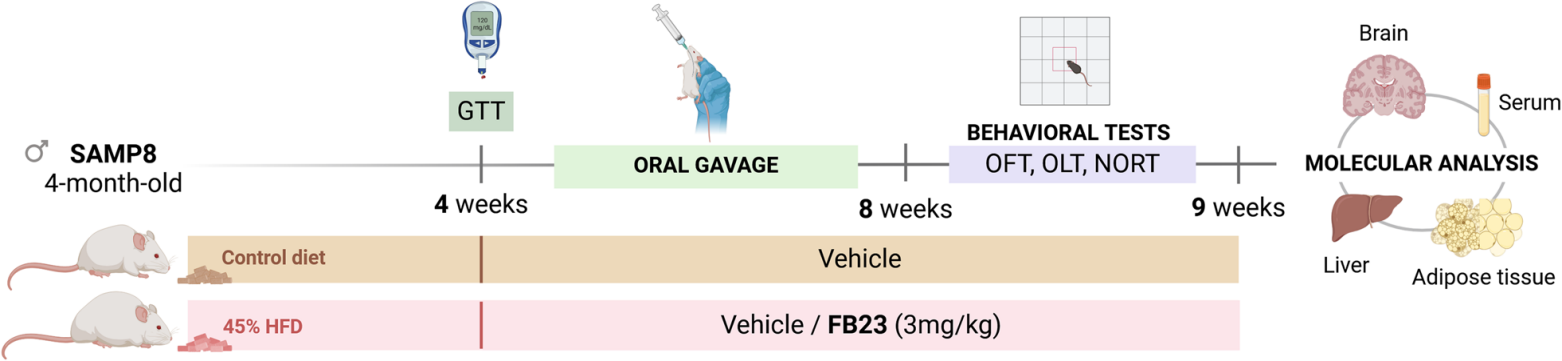
Experimental design scheme. 4-month-old SAMP8 male mice were fed a control diet or 45% kcal HFD for one month. GTT was performed before starting FB23 (3 mg/kg) or vehicle oral gavage treatment. After 4 weeks, behavioral tests were carried out. Molecular analysis was done in the brain, serum, liver, and white- and brown-adipose tissues

4-month-old male SAMP8 mice (n = 17) were used to perform behavioral and molecular studies. Animals had free access to food and water and were maintained under standard temperature conditions (22 ± 2 °C), controlled humidity, and a 12-h light/dark cycle (300 lx/0 lx). Animals were randomized into SAMP8 (n = 6), SAMP8 HFD (n = 5), and SAMP8 HFD FB23 (3 mg/kg) (n = 6) (Fig. 1). Furthermore, to cover all the molecular techniques, we divided the brain into two halves for further gene and protein determination, and Golgi staining. The SAMP8 group was fed a conventional diet (Inotiv, Madison, WI, USA, #2018 Teklad Global 18% Protein Rodent Diets). The high-fat diet (HFD) groups received a 45 kcal% fat (Research Diets Inc., NJ, USA, #D12451) for one month before the start and till the end of treatment. The exact composition of the conventional and HFD is shown in Tables S1 and S2, respectively. The animals were weighed weekly. The experimental groups received either a daily dose of vehicle (1% DMSO, Dimethyl sulfoxide) (Sigma-Aldrich, Steinheim, Germany, #D4540) with 20% w/v, (2-hydroxypropyl-β-cyclodextrin) (Atomole Scientific Co. Ltd., Wuhan, Hubei, China, #AT-20762) or a dose of 3 mg/kg per day of FB23 dissolved in vehicle, via oral gavage during the treatment period. After 4 weeks of treatment, behavioral and cognitive tests were performed. During this period and until sacrifice, the mice received FB23 or vehicle.

### Glucose tolerance test (GTT)

After 6 h of fasting, the mice received 2 g/kg of body weight glucose by intraperitoneal injection. Blood was collected from the tail vein for glucose determination after 0, 15, 30, 60, and 120 min.

### Behavioral and cognitive tests

#### Open field test (OFT)

The OFT was performed to assess the emotional changes and anxiety‐like behavior of the mice. A white polywood apparatus (50 × 50 × 25 cm) was used for the test. The bottom of the apparatus was divided into a central and a peripheral area (15 cm between the central area and the wall). Mice were placed in the center of the open field arena and allowed to explore the apparatus for 10 min. After the session, the mice were returned to their home cages, and the open field was cleaned with 70% ethanol and dried between sessions. Parameters assessed included time spent in the central and peripheral zones, number of rearing, grooming, defecation, and urination.

#### Object location test (OLT)

The OLT was performed to investigate the spatial memory of mice when exposed to a new location of an already-known object. This test is based on the spontaneous tendency of mice to spend more time exploring a novel object than a known object and to recognize when an object has been misplaced. The test was performed in a wooden box (50 × 50 × 25 cm), with three white walls and the last black. The animals were habituated to the empty outdoor area on the first day for 10 min. On the second day, two identical objects were placed in front of the black wall, equidistant from each other and from the wall. The objects were 10 cm high. The animals were brought into the open field arena and allowed to explore the objects and the surroundings for 10 min. The animals were then returned to their home cages and the OLT apparatus was cleaned with 70% ethanol. On the third day, an object was relocated in front of the opposite white wall to assess spatial performance of the mice. Trials were recorded with a camera placed above the open field area, and the total exploration time was determined by counting the time (in seconds) spent sniffing the object at the new location (TN) and the object at the old location (TO). The discrimination index (DI), which is defined as (TN − TO)/ (TN + TO), was calculated to assess the cognitive performance.

#### Novel object recognition test (NORT)

Short- and long-term memory was analyzed with NORT. The test was performed in a 90°, two‐armed, 25‐cm‐long, and 20‐cm‐high black maze. The light intensity in the middle of the field was 30 lx. The objects to be discriminated were plastic figures (object A, 5.25 cm high; and object B, 4.75 cm high). The mice were acclimatized to the device for 10 min on the 3 consecutive days (habituation phase). On day 4, they performed a 10-min acquisition trial (familiarization phase) in which they were placed in the maze and allowed to explore two identical novel objects (A + A or B + B) at the end of each arm. Objects A and B were counterbalanced to avoid a bias in object preference so that half of the animals in each experimental group were first confronted with object A and the other half with object B. For the analysis of long-term memory, object C was used as the novel object for both groups. The ten-minute retention trials (test phase) took place 2 h (short-term; A + B or B + A) and 24 h (long-term; A + C or B + C) after the habituation phase. During the test phase, one of the two identical objects was replaced by a novel one and the time spent exploring the new object (TN) and the old object (TO) was measured manually. The discrimination index (DI) was calculated as (TN − TO/TN + TO). Sniffing or touching objects with the nose and forepaws was considered exploration. The maze, surface and objects were cleaned with 70% ethanol between trials to eliminate olfactory cues.

### Biochemical and molecular experiments

#### Isolation of the tissues

The mice were euthanized by cervical dislocation 3 days after the last experiment was performed. Blood was collected in microtubes (approximately 0.03 ml per animal) (Sarstedt Inc Push Cap Micro Tube, 1.1 mL, Serum-Gel, #NC9436363), followed by a collection of the brain, liver, epididymal white adipose tissue (eWAT) and brown adipose tissue (BAT). The hippocampus and cortex were quickly dissected from the brain. All tissues were frozen in dry ice powder and stored at − 80 °C until use. Blood samples were centrifuged at 10000 g for 10 min at 4 ºC to obtain serum.

#### Measurement of serum leptin levels

According to the manufacturer's protocol, the collected serum samples (n = 4 mice per group) were used to determine serum leptin levels using the Mouse Leptin ELISA kit (Merck Millipore, #EZML-82 K).

#### Liver lipid content

Hepatic TG and total cholesterol (n = 3–4 mice per group) were extracted as described by Qu et al. (2007) and determined using a colorimetric assay kit (Spinreact, Girona, Spain, #1001311 and #1001091, respectively), according to the manufacturer’s protocol.

#### RNA extraction and determination of gene expression

Liver, eWAT and BAT tissues (n = 3–4 mice per group) were homogenized using the TissueLyser LT (QIAGEN, #85600). Total RNA was purified from mice samples using the Illustra RNAspin Midi RNA Isolation Kit (GE Healthcare, Cytiva, #11565065). cDNA was synthesized using the M-MLV reverse transcriptase (Invitrogen, USA, #28025013). Real-time PCR (qPCR) was performed using the LightCycler 480 SYBR Green I Master Kit (Roche, Mannheim, Germany, #04707516001) and measured with the Roche LightCycler 480 I Real-Time PCR System (Roche, Mannheim, Germany). The sequences of the oligonucleotides used are summarized in Table 1.

**Table 1:** SYBR Green primers sequences for liver, eWAT and BAT tissues gene expression determination by qPCR

gene	Forward sequence (5–3’)	Reverse sequence (5–3’)
*ARG1*	CTCCAAGCCAAAGTCCTTAGAG	AGGAGCTGTCATTAGGGACATC
*ATGL*	TGTAGGTGGCGCAAGACA	TGACCATCTGCCTTCCAGA
*BIP*	ACTTGGGGACCACCTATTCCT	ATCGCCAATCAGACGCTCC
*BMP8B*	ATGCGAGTCCGCTAAACG	GGCCCAGTAGCCATAGGAGT
*CATALASE*	GTGCATGCATGACAACCAG	TGAAGCGTTTCACATCTACAGC
*CIDEA*	AGAACTCCTCTGTGTCCACCA	GCCTGCAGGAACTTATCAGC
*CPT1B*	TGCCTTTACATCGTCTCCAA	GGCTCCAGGGTTCAGAAAGT
*DGAT2*	CCCTCAACACAGGCATTCG	GCACAGAGGCCACAGAAGTG
*EDEM*	AAGCCCTCTGGAACTTGCG	AACCCAATGGCCTGTCTGG
*FAS*	CAGATGATGACAGGAGATGGAA	CACTCACACCCACCCAGA
*FTO*	GACACTTGGCTTCCTTACCTG	CTCACCACGTCCCGAAACAA
*GLUT4*	GATGACCGTGGCTCTGCT	GCTCTGCCACAATGAACCA
*HSL*	CGCTCTCCAGTTGAACCAAG	GCGCTGGAGGAGTGTTTTT
*IL-1B*	TGTGAAATGCCACCTTTTGA	GGTCAAAGGTTTGGAAGCAG
*LEPTIN*	CAGGATCAATGACATTTCACACA	GCTGGTGAGGACCTGTTGAT
*MCP1*	GCAGGTGTCCCAAAGAAGCT	CAGCACAGACCTCTCTCTTGA
*MGL*	TGAGAAAGGCTTTAAGAACTGGG	GACCACCTGTAGTGATGTGGG
*PDI*	ACCTGCTGGTGGAGTTCTATG	CGGCAGCTTTGGCATACT
*PEPCK*	GTCAACACCGACCTCCCTTA	CCCTAGCCTGTTCTCTGTGC
*PGC1A*	GAAAGGGCCAAACAGAGAGA	GTAAATCACACGGCGCTCTT
*PLIN2*	CCAGGACAGTCTGGCATGT	GAGTCCCACTGTGTTGAGCA
*PPARA*	CACGCATGTGAAGGCTGTAA	CAGCTCCGATCACACTTGTC
*PPARG*	CGCTGATGCACTGCCTATGA	AGAGGTCCACAGAGCTGATTCC
*PRDM16*	CCTAAGGTGTGCCCAGCA	CACCTTCCGCTTTTCTACCC
*RPL32*	CTGGAGGTGCTGCTGATGT	GGGATTGGTGACTCTGATGG
*SOD*	CAGGACCTCATTTTAATCCTCAC	CCCAGGTCTCCAACATGC
*SREBP*	CGGAAGCTGTCGGGGTAG	GGCCAGAGAAGCAGAAGAGA
*TNFA*	TCGGGGTGATCGGTCCCCAA	TGGTTTGCTACGACGTGGGCT
*UCP1*	GGCCTCTACGACTCAGTCCA	TAAGCCGGCTGAGATCTTGT

Isolation of total RNA from brain samples (n = 3–4 mice per group) was performed using TRItidy G^™^ reagent (ITW Reagents, Panreac AppliChem, #A4051) according to the manufacturer’s instructions. Reverse transcription polymerase chain reaction (RT-PCR) was then performed as follows: 2 μg of messenger RNA (mRNA) was reverse transcribed using the High-Capacity cDNA Reverse Transcription Kit (Applied Biosystems^™^, Vilnius, Lithuania, #4368813). qPCR was performed with the StepOnePlus^™^ Real-Time PCR System (Applied Biosystems^™^, Vilnius, Lithuania, #4376600), using the SYBR Green PCR Master Mix (Applied Biosystems^™^, Vilnius, Lithuania, #K0253). Briefly, each reaction mixture consisted of 6.75 μL of cDNA (at a concentration of 2 μg), 0.75 μL of each primer (at a concentration of 100 nM), and 6.75 μL of SYBR Green PCR Master Mix (2X). The primer sequences are listed in Table 2.

**Table 2:** SYBR Green primers sequences for brain tissue gene expression determination by qPCR

Gene	Forward sequence (5–3’)	Reverse sequence (5–3’)
*FTO*	GACACTTGGCTTCCTTACCTG	CTCACCACGTCCCGAAACAA
*YTHDC1*	GCGTAGGAAGCTGAGTGGAG	TCCCCATCTTTCTCCTCCCG
*YTHDC2*	ACAAAACATGCTGTTAGGAGCC	CCCGCTTGTCTTGCTCATTTC
*YTHDF1*	GGGACAAGTGGTTCTCAGGG	TCCCCAATCTTCAGGCCAAC
*YTHDF2*	CAGCTCTCAGTCCAGCAACA	AGTAGATCCAGAACCCGCCT
*YTHDF3*	CAGAGACCTAAAGGGCAAGGA	CATGCTGCTTCCCCAAGAGA
*IL-6*	ATCCAGTTGCCTTCTTGGGACTGA	TAAGCCTCCGACTTGTGAAGTGGT
*MCP1*	CCCACTCACCTGCTGCTACT	TCTGGACCCATTCCTTCTTG
*INOS*	GGCAGCCTGTGAGACCTTTG	GAAGCGTTTCGGGATCTGAA
*CHOP*	TTGAAGATGAGCGGGTGGCAG	CACGTGGACCAGGTTCTCTCTC
*ATF3*	TTGCTAACCTGACACCCTTTG	CGGTGCAGGTTGAGCATGTA
*ATF6*	TTTCAGGGCAGGGCCATT	CCCGGGACAAACAGGTCTT
*BIP*	CTGGGTACATTTGATCTGACTGG	GCATCCTGGTGGCTTTCCAGCCATTC
*CATALASE*	AGGAGGCGGATCTAGCCTTA	TGTCAAGAAAGGGGTGTCGT
*MMP-2*	ACCCAGATGTGGCCAACTAC	TACTTTTAAGGCCCGAGCAA
*MMP-9*	CCTGGAACTCACACGACATCTTC	TGGAAACTCACACGCCAGAA
*IGF-1*	CGAATGTTCCCCCAGCTGTT	GGCAGGGATAATGAGGCGAA
*PI3K*	ACCCAGCAACAGAAAAATGG	GCGCTGTGAATTTAGCCTTC
*PTEN*	CGGCAGCATCAAATGTTTCAG	AACTGGCAGGTAGAAGGCAACTC
*LEPTIN RECEPTOR*	CTGCACTTAACCTGGCATATCCA	GGCTCCAGCAGGTGAGAGAA
*ARC*	GCCCCCAGCAGTGATTCATA	GGAACCCATGTAGGCAGCTT
*CFOS*	CCCGTAGACCTAGGGAGGAC	CAATACACTCCATGCGGTTG
*STAT3*	CCGAACCCCATATCGTCTGAA	TGTTCCCCTTTGCCTCCCTTC
*BDNF*	TGCGAGTATTACCTCCGCCAT	TCACGTGCTCAAAAGTGTCAG
*NGF*	GGAGCGCATCGAGTGACTT	CCTCACTGCGGCCAGTATAG
*VGF*	GTCAGACCCATAGCCTCCC	CTCGGACTGAAATCTCGAAGTTC
*SCG2*	CACCTACCTGAGAAGGAATTTGC	GACCAGGTGCCTTGGGATAC
*Β-ACTIN*	CAACGAGCGGTTCCGAT	GCCACAGGTTCCATACCCA

For all the studies, RNA yield, purity, and quality were determined using a NanoDrop^™^ ND-1000 (Thermo Fisher Scientific, Waltham, MA, USA). Samples were also tested in an Agilent 2100B Bioanalyzer (Agilent Technologies, Santa Clara, CA, USA) to determine the RNA integrity. RNAs with a 260/280 ratio number of 1.8-2and a RIN number of 7.5, respectively, were selected. The comparative cycle threshold (Ct) (ΔΔCt) method was used to analyze the data, using the housekeeping gene *β-actin* level in brain tissue and *rpl32* in liver, eWAT and BAT to normalize for differences in sample loading and preparation. Each sample (n = 3–4) was analyzed in duplicate, and the results represented the *n*-fold difference in transcript levels between the different groups.

#### Quantification of m6A RNA methylation

Total RNA extraction from brain tissue (n = 3–4 mice per group) was performed using TRItidy G^™^ reagent, and RNA yield, purity, and quality were determined, as described in the previous section. Quantification of m6A RNA methylation was performed using the EpiQuik^™^ m6A RNA Methylation Quantification Kit (Colorimetric) (Epigentek, #P-9005), according to the manufacturer’s protocol. The absorbance for each sample (200 ng RNA) was measured at 450 nm in a microplate spectrophotometer, and the optical density was considered proportional to the amount of m6A.

#### Western blotting

Brain tissue samples (n = 3–4 mice per group) were homogenized in lysis buffer (Tris HCl pH 7.4 mM, NaCl 150 mM, EDTA 5 mM and 1X-Triton X-100) containing EDTA-free Protease inhibitor cocktail (Roche, Mannheim, Germany, #11836170001), and Phosphatase inhibitor cocktail II (Sigma-Aldrich, Steinheim, Germany, #P5726), and protein concentration was determined by the Bradford method. Aliquots of 15 µg of protein were separated by sodium dodecyl sulfate–polyacrylamide gel electrophoresis (SDS-PAGE) (8–15%) and transferred into polyvinylidene difluoride (PVDF) membranes (Merck-Millipore, Burlington, MA, USA). Then, the membranes were blocked in 5% Bovine Serum Albumin (BSA) in 0.1% Tris-buffered saline with Tween 20 (TBS-T) for one hour at room temperature, followed by overnight incubation at 4 °C with the primary antibodies listed in Table 3. The next day, the membranes were washed with TBS-T and incubated with the secondary antibodies for one hour at room temperature. A chemiluminescence-based detection kit was used to view the immunoreactive proteins following the manufacturer’s protocol (ECL Kit; Millipore, Burlington, MA, USA), and digital images were acquired using an Amersham Imager 680 (BioRad, Hercules, CA, USA). Then, semiquantitative analyses were carried out using Image Lab software 6.1 (Bio-Rad). The results were expressed in arbitrary units (AUs), with the control protein levels set as 100%. The results for protein quantification were normalized to the control protein levels (glyceraldehyde-3-phosphate dehydrogenase, GAPDH).

**Table 3:** Antibodies used for brain tissue protein determination by Western Blotting

Antibody	Host	Source/catalog	WB dilution
P-AKT (SER473)	Rabbit	Cell signaling/#9271	1:1000
AKT	Rabbit	Cell signaling/#9272	1:1000
P-NFKB P65	Mouse	Santa Cruz/sc-166748	1:1000
NFKB P65	Rabbit	Cell signaling/#8242	1:1000
GAPDH	Mouse	Millipore/MAB374	1:2000
Goat-anti-mouse HRP conjugated		BioRad/170-5047	1:5000
Goat-anti-rabbit HRP conjugated		BioRad/170-6515	1:5000

#### Golgi staining, dendritic length and spine density

The SAMP8 mice were euthanized by cervical dislocation, and the whole brain was removed from the skull and separated into two halves, one of them, for performing Golgi staining (n = 3). Then, the Golgi staining protocol was performed using the FD Rapid GolgiStain™ Kit (D Neurotechnologies, Inc., #PK401) according to the manufacturer’s instructions. Images of neurons for the dendritic branching analysis were captured using a Leica Thunder microscope (Leica Thunder Imager; Leica Microsystems) taken at 40 × magnification. Neurite length and neuron complexity were measured using NeuronJ macros and Advanced Sholl Analysis. The number of intersections (branch points) within concentric circles with a radius of 10 µm was calculated and compared between groups. Images of neurons for the spine density analysis were taken at 63 × magnification of the oil objective. Spine density was expressed as the number of spines per 50 μm dendrite, at a maximum distance of 150 µm from the soma.

### Statistical analysis

The data were analyzed using GraphPad Prism Version 9 software (GraphPad Software, San Diego, CA, USA). The group size varied depending on the power analysis. Statistical analysis was performed for studies where the size of each group was n = 5–6 samples per group for in vivo studies and n = 3–4 independent replicates for in vitro studies. Results were expressed as mean ± standard error of the mean (SEM). A normality test was performed to ensure that parametric tests could be applied. Means were compared using one-way ANOVA followed by Tukey’s post hoc analysis. Comparison between two groups was performed using a two-tailed Student’s *T*-test for independent samples. Statistical significance was assumed if the p-values were < 0.05. Statistical outliers were identified using Grubbs’ test and removed from the analysis. A blind analysis was performed for the behavioral tests.

## Results

### HFD increases body weight and glucose intolerance in SAMP8 mice

**Fig. 2:**
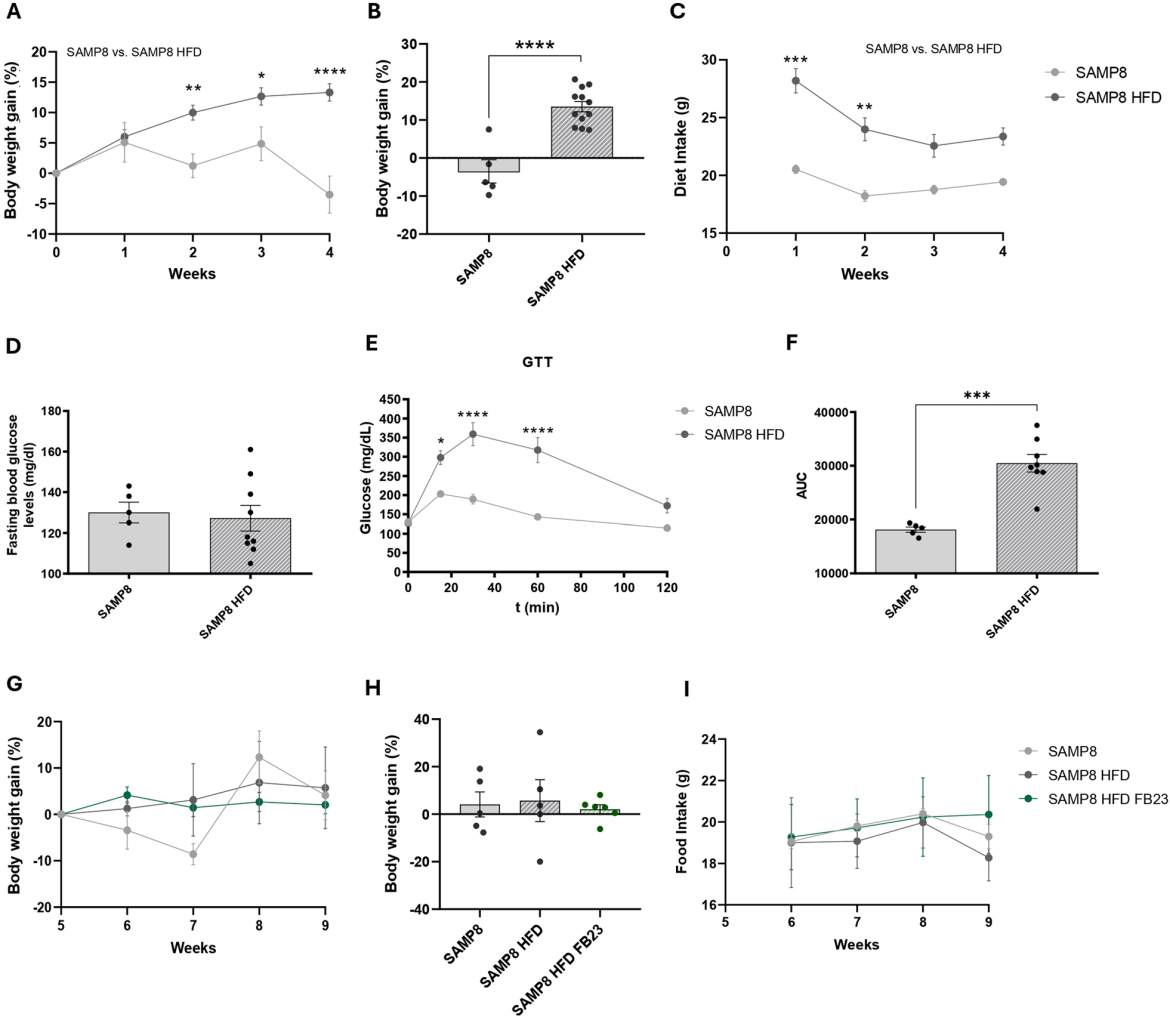
Body weight and glucose intolerance analysis in SAMP8 HFD mice. Body weight gain is shown as a percentage normalized to the initial weight **A** per week **B** at the end, and **C** food intake before treatment. **D** Fasting blood glucose values in mg/dL, **E** Glucose Tolerance Test (GTT), and **F** Area Under the Curve (AUC) in SAMP8 mice and SAMP8 mice fed a high-fat diet (HFD). Values are expressed as mean ± SEM. Groups were compared by two-way ANOVA analysis followed by Sidak’s post-hoc test (**A**, **C**, **E**). Groups were compared using unpaired Student’s t-test (**B**, **D**, **F**). n = 5–12 per group; Body weight gain is shown as a percentage normalized to the initial weight **G** per week and **H** at the end, and **I** diet intake follow-up after the treatment. SAMP8 HFD FB23 group is presented after the treatment. Values are expressed as mean ± SEM. Groups were compared using a two-way ANOVA analysis followed by Sidak’s post-hoc test (**G**, **I**). Groups were compared by unpaired one-way ANOVA analysis followed by Tukey’s post-hoc test (**H**). n = 5–12 per group; *p < 0.05; **p < 0.01; ***p < 0.001; ****p < 0.0001

Metabolic disruption is known to be one of the main factors contributing to age-related cognitive decline and neurodegenerative diseases (Amidfar et al. 2024). Herein, we demonstrated that, after one month of HFD feeding, SAMP8 mice showed a significantly increased body weight compared to the SAMP8 control diet group (Figs. 2A, B). Interestingly, HFD-fed mice showed a higher consumption of diet compared to SAMP8 control mice (Fig. 2C). Regarding glucose metabolism, although animals showed no differences in fasting blood glucose levels (Fig. 2D), HFD-fed mice were significantly affected in GTT analysis, showing an impaired glucose tolerance in SAMP8 HFD mice (Fig. 2E, F). For carrying out FB23 treatment, SAMP8 HFD mice were randomly divided into control and treated groups for the following four weeks. No differences were seen in body weight among groups (Fig. 2G, H), nor in the amount of diet intake (Fig. 2I).

### Increased leptin levels and characterization of genes involved in lipid metabolism, oxidative damage, and inflammation in eWAT after FTO inhibition in SAMP8 HFD mice

**Fig. 3:**
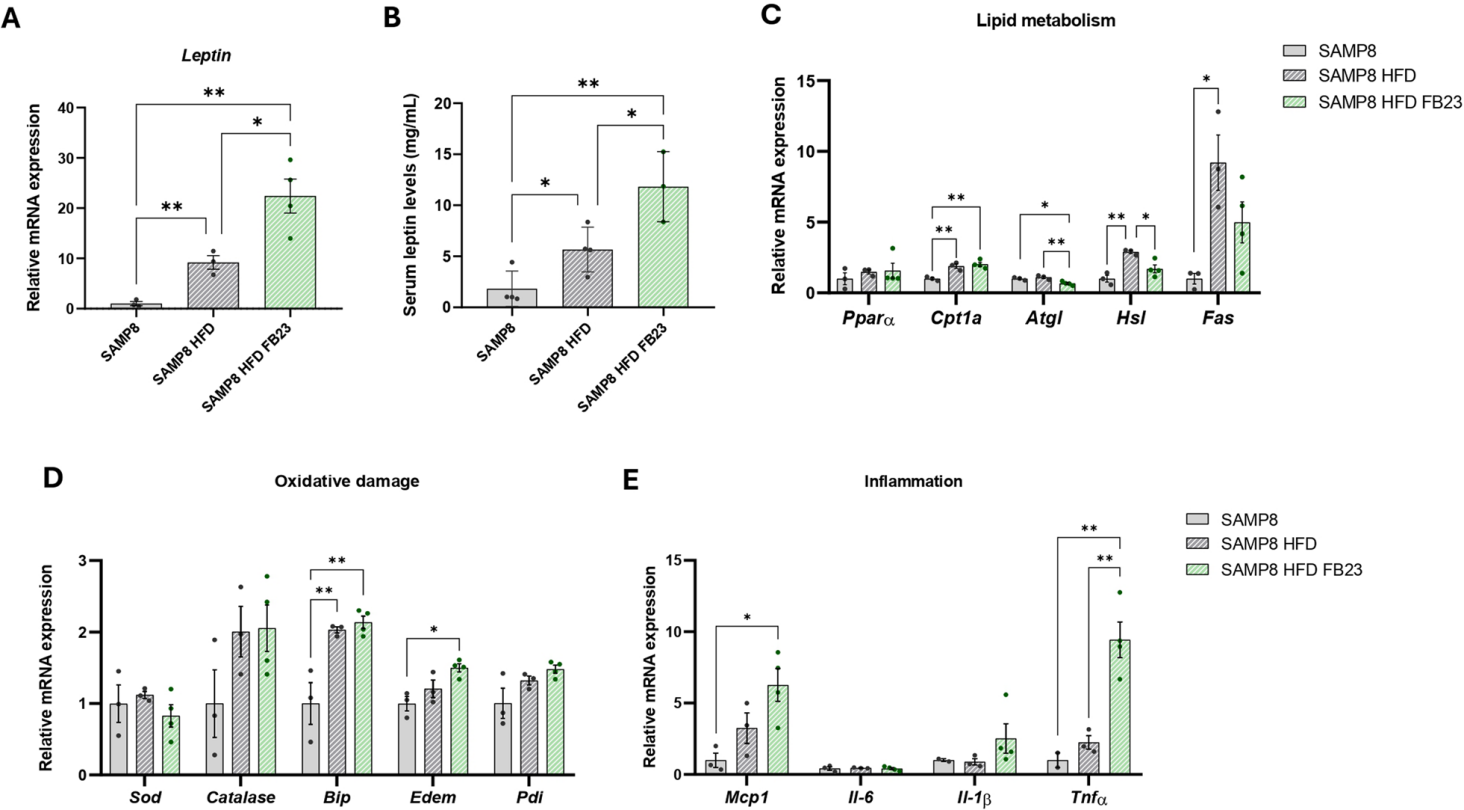
Characterization of lipid metabolism in eWAT. **A** Representative eWAT gene expression of *Leptin*. **B** Serum leptin levels expressed in mg/mL. **C** Representative eWAT gene expression of *Pparα, Cpt1α, Atgl, Hsl* and *Fas*, **D** *Sod, Catalase, Bip, Edem* and *Pdi*, and **E** *Mcp1, Il-6, Il-1β* and *Tnfα*. Relative mRNA expression levels were determined by real-time PCR. Values are expressed as mean ± SEM. Groups were compared by one-way ANOVA analysis followed by Tukey’s post-hoc test. If necessary, groups were compared using a two-tailed Student’s t-test. n = 3–4 per group. *p < 0.05; **p < 0.01

Adipocytes produce the adipokine leptin and maintain a homeostatic level in the blood that is proportional to the amount of adipose tissue, which correlates the animals’ food intake with energy storage (Saxton et al. 2023). Consistent with the observed trend in body weight, *leptin* gene expression was increased in eWAT in HFD-fed mice compared to the SAMP8 control group (Fig. 3A). Serum leptin levels were also significantly increased in HFD mice (Fig. 3B). As for the effect of FB23 treatment, gene expression of *leptin* was further increased in eWAT from HFD-treated mice compared with the HFD control group (Fig. 3A), suggesting possible enhanced signaling of the adipokine throughout the body. In addition, lipid metabolism in eWAT was also altered after HFD and FTO inhibition. Although no differences were observed in the expression of *peroxisome proliferator-activated receptor alpha (Pparα)* receptor, its target gene *carnitine palmitoyl-transferase 1a (Cpt1a),* which transports long-chain fatty acids to the mitochondria for oxidation (Liang 2023), was significantly increased in the HFD groups compared with the SAMP8 control mice (Fig. 3C). The mRNA levels of enzymes that break down triglycerides (TG), such as *adipose triglyceride lipase (Atgl)* and *hormone-sensitive lipase (Hsl),* which were increased in the SAMP8-HFD control mice compared with the SAMP8 control group, were significantly attenuated after FTO inhibition (Fig. 3C), which suggest a reduced amount of metabolic intermediates such as fatty acids (FAs) (Brejchova et al. 2021). Following the same pattern, *Fatty acid synthetase (Fas),* which regulates de novo lipogenesis, was significantly increased in the SAMP8-HFD control mice (Fig. 3C).

Dysregulation of lipid metabolism leads to increased oxidative damage and an inflammatory environment in eWAT tissue. Our data showed no changes in *superoxide dismutase (Sod)*, although other neutralizers of reactive oxygen species, such as *binding immunoglobulin protein (Bip)* and *Endoplasmic reticulum* (*ER) degradation enhancing alpha-mannosidase-like protein (Edem),* were significantly increased in SAMP8 HFD FB23-treated mice (Fig. 3D). *Catalase* and *protein disulfide isomerase (Pdi)* followed the same pattern, although the differences were not significant (Fig. 3D). Inflammatory markers were also increased after FTO inhibition, showing a significant increase in *monocyte chemoattractant protein 1 (Mcp1)*, which protects neurons from damage caused by oxygen–glucose deprivation (Madrigal et al. 2009), and *tumor necrosis factor-alpha (Tnfα)*, which may exert a neuroprotective effect by stimulating neuronal plasticity (Zhang et al. 2011), in SAMP8 HFD-treated mice (Fig. 3E). The pro-inflammatory cytokines *interleukin-6 (Il-6)* and *interleukin-1β (Il-1β)* were not altered in this study (Fig. 3E). Overall, the inflammatory response of adipocytes in FB23-treated mice appeared to be activated.

### Evaluation of hepatic lipid content and genes involved in glucose metabolism, lipolysis, lipogenesis, and oxidative damage after FB23 treatment in SAMP8 HFD mice

**Fig. 4:**
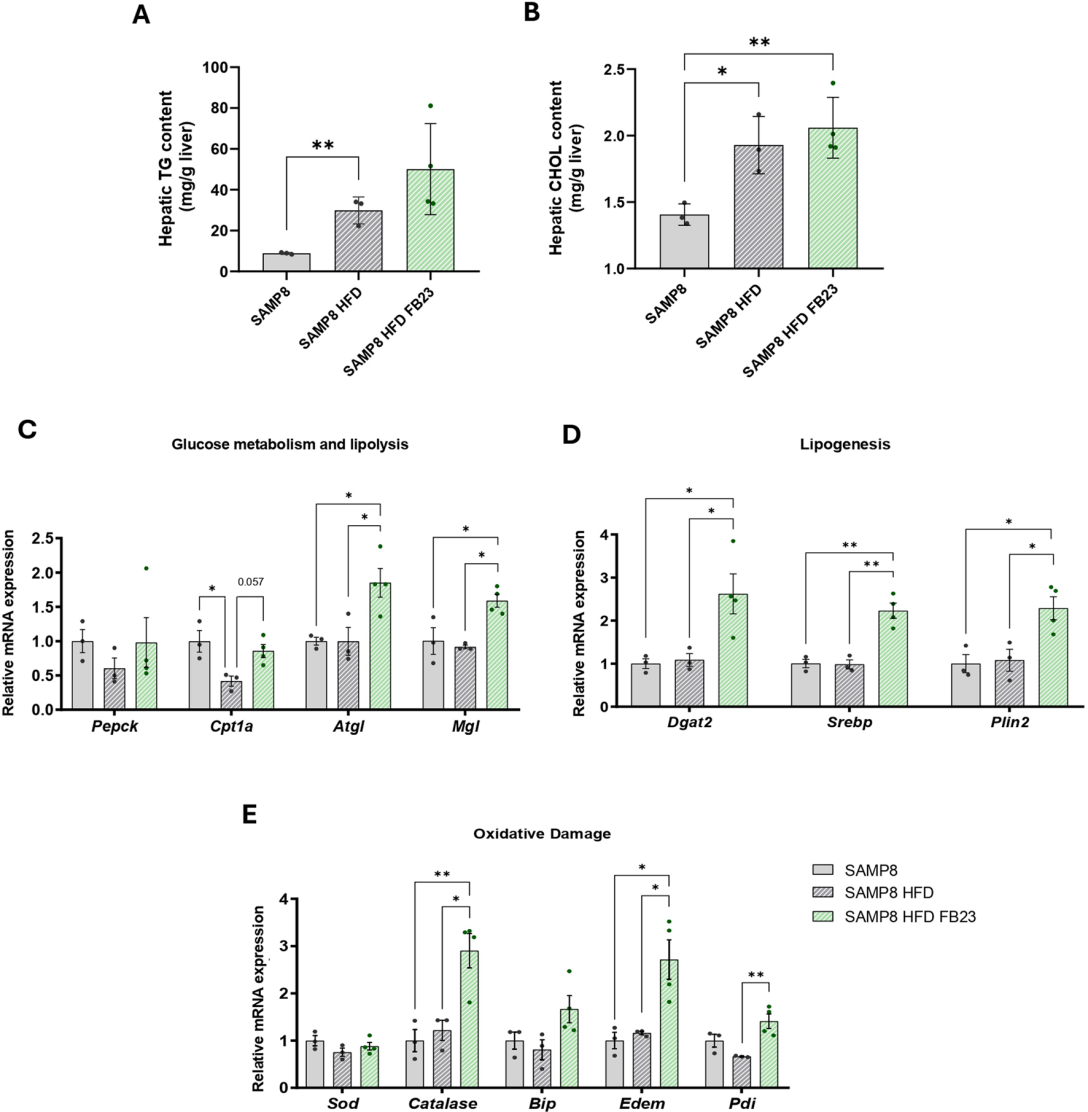
Characterization of lipid and glucose metabolism in liver. Hepatic **A** triglycerides (TG) content and **B** cholesterol content (CHOL) in mg/g liver tissue. Relative liver gene expression of **C** *Pepck, Cpt1α, Atgl* and *Mgl*, **D** *Dgat2, Srebp* and *Plin2*, and **E** *Sod, Catalase, Bip, Edem* and *Pdi*. Relative mRNA expression levels were determined by real-time PCR. Values are expressed as mean ± SEM. Groups were compared by one-way ANOVA analysis followed by Tukey’s post-hoc test. If necessary, groups were compared using a two-tailed Student’s t-test. n = 3–4 per group. *p < 0.05; **p < 0.01

To investigate hepatic lipid accumulation in HFD mice, we analyzed both TG and cholesterol levels in the liver. We confirmed that the SAMP8-HFD control mice had significantly higher hepatic TG and cholesterol levels compared to the SAMP8 control group (Fig. 4A, B). Moreover, we observed that this increase in lipid content was no longer enhanced after FTO inhibition treatment (Fig. 4A, B). As a consequence of liver lipid accumulation, alterations in genes involved in glucose and lipid metabolism were detected in the HFD control mice. The expression of genes related to lipid catabolism such as *Cpt1a, Atgl* and *monoacylglycerol lipase (Mgl)* increased significantly after FTO inhibition in SAMP8-HFD mice, with a slight downward trend in the first compared to SAMP8 mice fed a normal diet (Fig. 4C). *Phosphoenolpyruvate carboxykinase 1 (Pepck)*, a gluconeogenesis checkpoint in the liver, was not altered by treatment or diet (Fig. 4C). At the same time, the expression of several genes involved in lipogenesis, including *diacylglycerol O-acyltransferase 2 (Dgat2), sterol regulatory element-binding protein (Srebp),* and *perilipin 2 (Plin2,)* were also significantly increased in FB23-treated mice compared with HFD-fed SAMP-8 mice (Fig. 4D). Overall, the increase in lipolysis and lipogenesis observed after FTO inhibition suggest a compensatory mechanism of the liver against the metabolic and energetic imbalance caused by HFD uptake. Gene expression of attenuators of oxidative damage was increased after FTO inhibition. *Catalase, Edem* and *Pdi* showed a significant difference between the SAMP8-HFD control group and the FB23-treated group (Fig. 4E). *Sod* and *Bip* were not significantly altered (Fig. 4E).

### Inhibition of FTO showed minor changes in genes involved in thermogenesis, glucose and lipid metabolism, and oxidative damage in the BAT of SAMP8 HFD mice

**Fig. 5:**
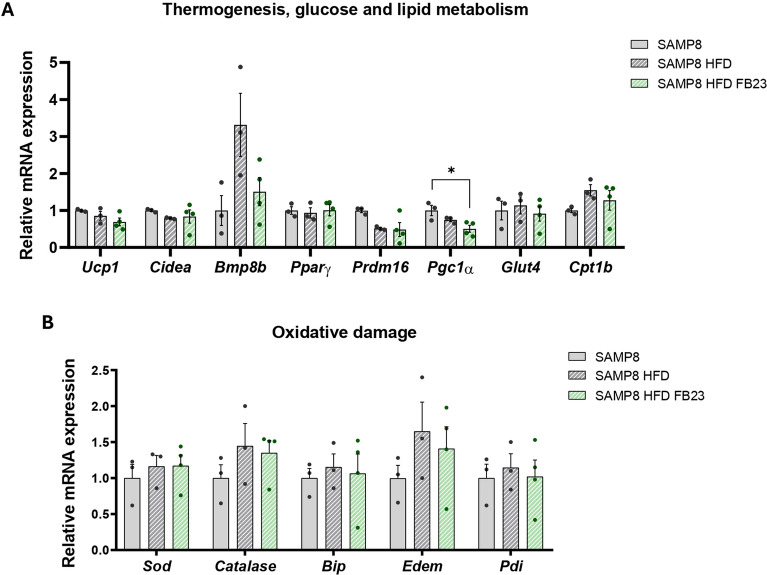
Minor changes in BAT metabolic function. Representative BAT gene expression of **A** *Ucp1, Cidea, Bmp8b, Pparγ, Prdm16, Pgc1α, Glut4* and *Cpt1b*, and **B** *Sod, Catalase, Bip, Edem* and *Pdi*. Relative mRNA expression levels were determined by real-time PCR. Values are expressed as mean ± SEM. Groups were compared by one-way ANOVA analysis followed by Tukey’s post-hoc test. n = 3–4 per group. *p < 0.05

Brown adipocytes are the main regulators of thermal homeostasis and are characterized by a larger number of mitochondria compared to white adipocytes. However, it is known that both the amount and the function of BAT decrease with age (Li et al. 2024). *Peroxisome proliferator-activated receptor gamma coactivator 1 alpha (Pgc1α),* the master regulator of mitochondrial biogenesis, was significantly decreased after FB23 treatment in BAT samples from SAMP8 HFD mice (Fig. 5A). Many other regulators involved in thermogenesis and glucose and lipid metabolism in BAT, such as *uncoupling protein 1 (Ucp1), cell death inducing DFFA like effector A (Cidea), bone morphogenetic protein 8B (Bmp8b), Pparγ, PR domain-containing protein 16 (Prdm16), glucose transporter 4 (Glut4)* and *Cpt1b* were not significantly altered (Fig. 5A). No differences were observed for oxidative damage markers such as *Catalase, Bip, Edem* and *Pdi* (Fig. 5B). Our data suggests a possible decreased synthesis and oxidative activity of mitochondria in BAT, which could be explained by accelerated aging in our mouse model.

### FTO inhibition modulates m6A RNA methylation and neuroinflammatory markers in SAMP8 HFD mice

**Fig. 6:**
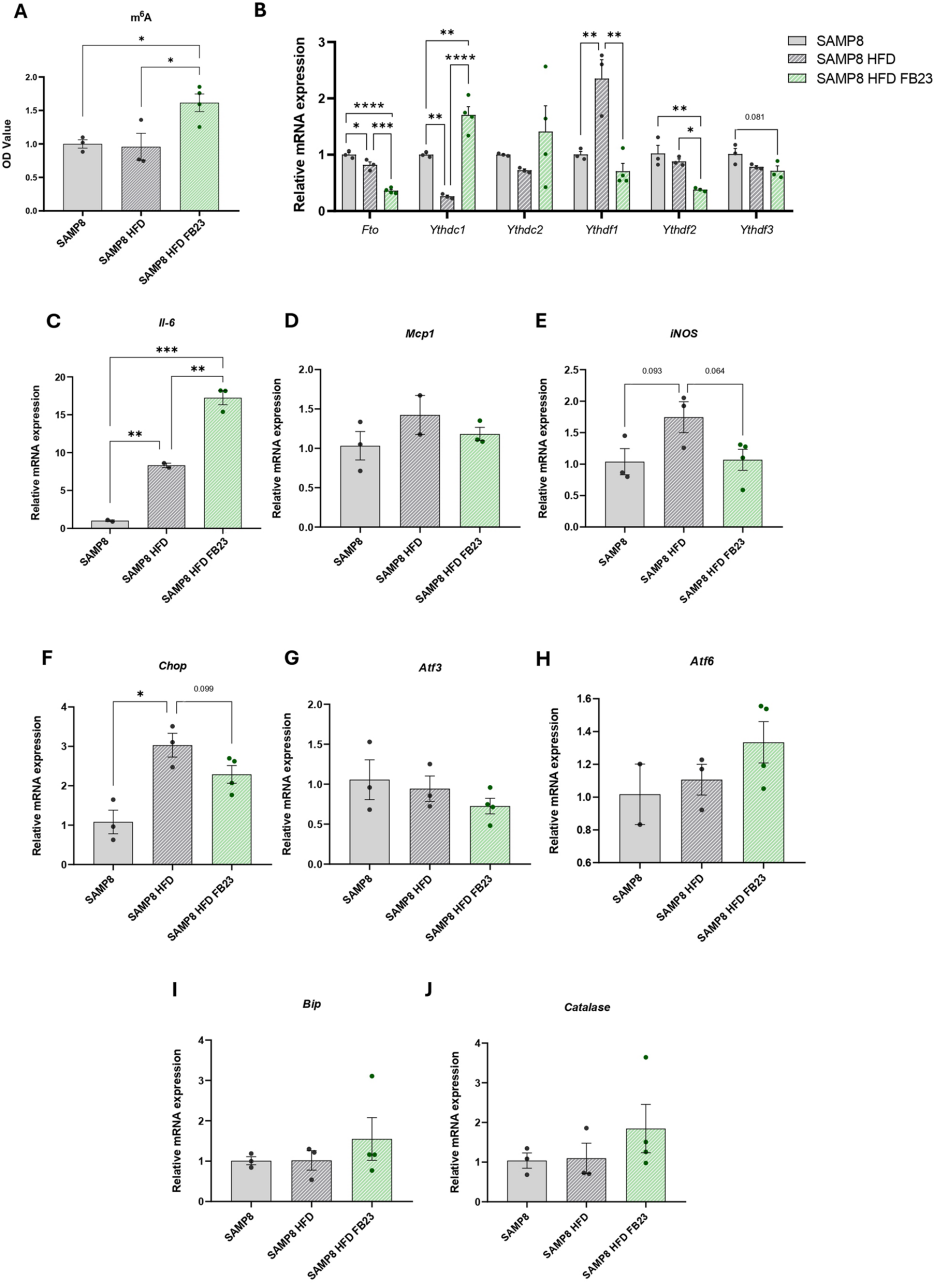
Modulation in m6A methylation and neuroinflammatory markers in SAMP8 HFD mice after FB23 treatment. **A** Quantification of m6A RNA methylation levels in the brain. OD values were determined using the EpiQuik™ m6A RNA methylation Quantification Kit. Representative gene expression in the brain of **B** m6A-regulatory enzymes: *Fto, Ythdc1, Ythdc2, Ythdf1, Ythdf2* and *Ythdf3*, **C** *Il-6*, **D** *Mcp1*, **E** *iNos*, **F** *Chop*, **G** *Atf3*, **H** *Atf6*, **I** *Bip* and **J** *Catalase*. Relative mRNA expression levels were determined by real-time PCR. Values are expressed as mean ± SEM. Groups were compared by one-way ANOVA analysis followed by Tukey’s post-hoc test. n = 3–4 per group (outliers: n = 1 in the SAMP8 HFD group for *Mcp1* ratio values and n = 1 in the SAMP8 group for *Atf6* ratio values); *p < 0.05; **p < 0.01; ***p < 0.001; ****p < 0.0001

m6A RNA methylation is highly present in the brain, and changes in its levels are associated with neurodegeneration (Zhang et al. 2022). We found that m6A was significantly increased after treatment with FB23 in the brain of SAMP8 HFD mice, even compared to the SAMP8 control groups (HFD and normal diet) (Fig. 6A). Brain *Fto* decreased significantly after treatment (Fig. 6B). m6A-regulatory enzymes, known as readers, which are proteins responsible for activating downstream m6A signaling, were also found to be altered: *YTH N6-methyladenosine RNA-binding protein (Ythd) C1 (Ythdc1)* and *Ythdc2* were increased after FTO inhibition, while *Ythdf1, Ythdf2* and *Ythdf3* decreased (Fig. 6B), suggesting a complex reorganization of m6A-dependent RNA regulation, that may have implications for various cellular processes and neurodegenerative diseases.

Neuroinflammation is one of the main factors contributing to cognitive decline (DiSabato et al. 2016). Recent studies have shown that m6A modification plays a crucial role in the immune response, especially in disease (Luo et al. 2021). In this study, *Il-6* was significantly increased in the brain of FB23-treated mice, compared to SAMP8 control mice and SAMP8-HFD control mice (Fig. 6C). In addition, *Mcp1*, the most highly expressed chemokine in brain inflammation, and *inducible nitric oxide synthase (iNOS)*, a major producer of reactive oxygen species, showed no significant differences after treatment (Fig. 6D, E). Closely related stress regulators of the ER were also altered after FTO inhibition. *C/EBP homologous protein (Chop),* which was significantly increased in SAMP8-HFD mice, decreased after FB23 treatment (Fig. 6F). *Activating transcription factor 3 (Atf3)* tended to decline, while *Atf6* increased (Figs. 6G, H). Both mitigators of oxidative damage, *Bip* and *Catalase*, showed no differences after treatment with FB23 (Fig. 6I, J). Overall, FTO inhibition played a key role in the neuroinflammation present in SAMP8 HFD mice.

### FTO inhibition modulates IGF1 downstream signaling in SAMP8 HFD mice

**Fig. 7:**
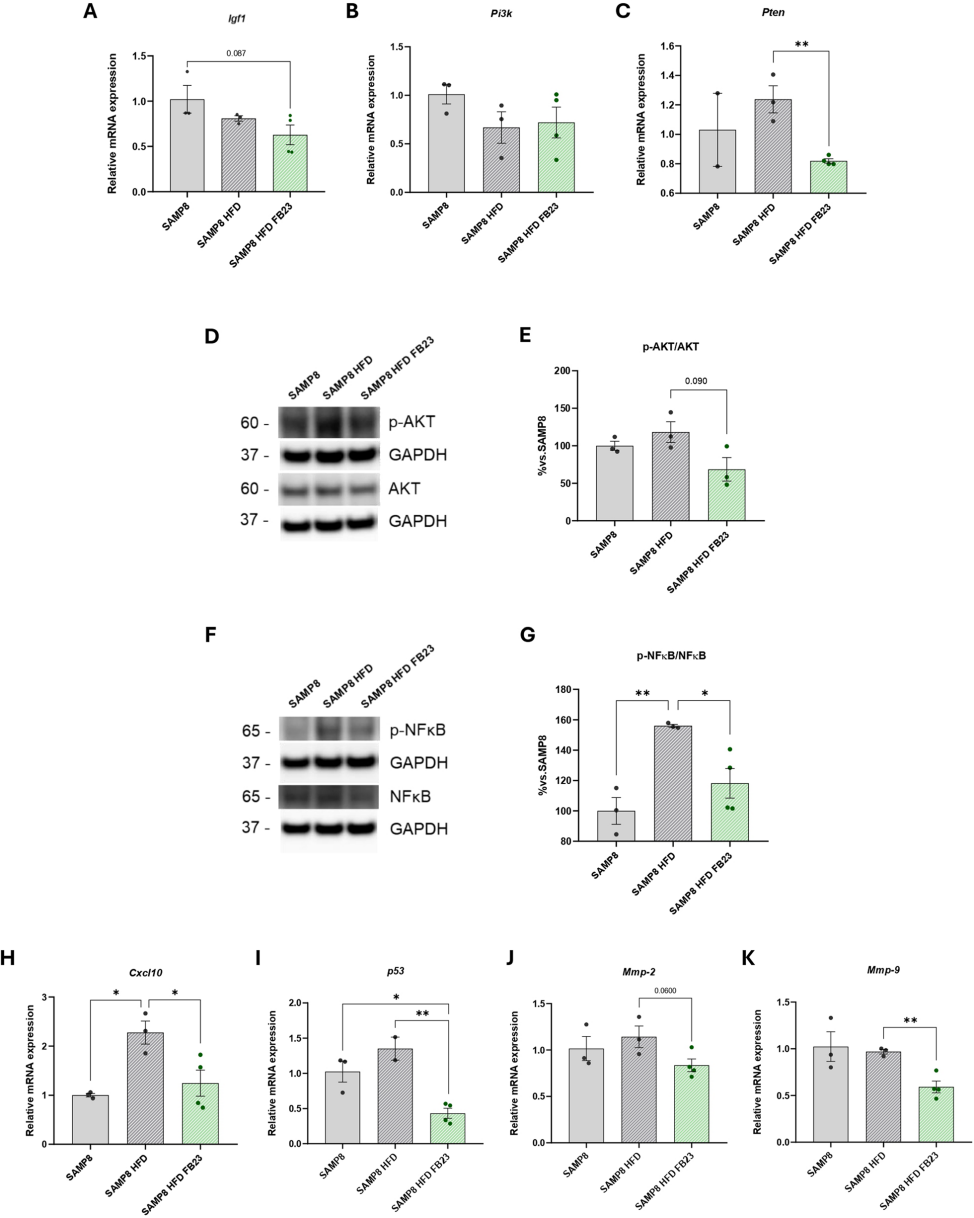
Decrease in IGF1/AKT activation pathway in SAMP8 HFD mice after FB23 treatment. Representative gene expression in the brain of **A** *Igf1*, **B** *Pi3k* and **C** *Pten*. Immunoblot and quantification of **D**, **E** p-AKT/AKT ratio levels and **F**, **G** p-NFKB/NFKB ratio levels. Representative gene expression of (**H**) *Cxcl10,* (**I**) *p53,* (**J**) *Mmp-2* and (**K**) *Mmp-9*. Relative mRNA expression levels were determined by real-time PCR. Protein ratio levels were determined by Western blotting. Values are expressed as mean ± SEM. Groups were compared by one-way ANOVA analysis followed by Tukey’s post-hoc test. n = 3–4 per group (outliers: n = 1 in the SAMP8 group for *Pten* gene levels; n = 1 in the SAMP8 HFD FB23 group for p-AKT/AKT protein levels; n = 1 in the SAMP8 HFD group for *p53* gene levels); *p < 0.05; **p < 0.01

The IGF1 signaling pathway is known to regulate brain aging by promoting neuronal differentiation and survival (Wrigley et al. 2017). A decreasing trend of *insulin-like growth factor 1* (*Igf1)* gene expression was observed in SAMP8 HFD FB23 mice (Fig. 7A). No changes were observed for *phosphoinositide 3-kinase (Pi3k)* between groups (Fig. 7B), while a significant decrease was observed for *phosphatase and tensin homolog (Pten)*, its negative regulator, after FTO inhibition (Fig. 7C). Following IGF1/PI3K/AKT pathway (Li et al. 2023), a reduction in both phospho-protein kinase B (p-AKT)/AKT and phospho-nuclear factor kappa-light-chain-enhancer of activated B cells (p-NFκB)/NFκB ratio levels was observed in SAMP8 HFD FB23 mice (Fig. 7D, E, F, G). Downstream targets of NFκB protein, such as *C-X-C motif chemokine ligand 10 (Cxcl10)* and *transformation-related protein 53 (p53),* were also found to significantly decrease in SAMP8 HFD mice after FTO inhibition (Fig. 7H, I). Apart from the pro-inflammatory markers, matrix metalloproteases (MMPs) were also found to be altered after IGF1 pathway downregulation. MMPs are involved in the regulation of plastic changes at the level of the synapses by processing extracellular matrix proteins (Almeida et al. 2022). We investigated two MMPs that exhibit gelatinase activity. Both decreased after FTO inhibition in the brain of SAMP8-HFD mice, with a clear trend for *matrix metalloproteinase-2 (Mmp-2)* and a significant difference for *Mmp-9* (Fig. 7J, K). Recently, a coordinated forward loop was found between MMPs and IGF1 as promoters for its release from the membrane (Kok and Barton 2021).

### FTO inhibition enhances leptin signaling and promotes synaptic plasticity in the cortical tissue of SAMP8 HFD mice

**Fig. 8:**
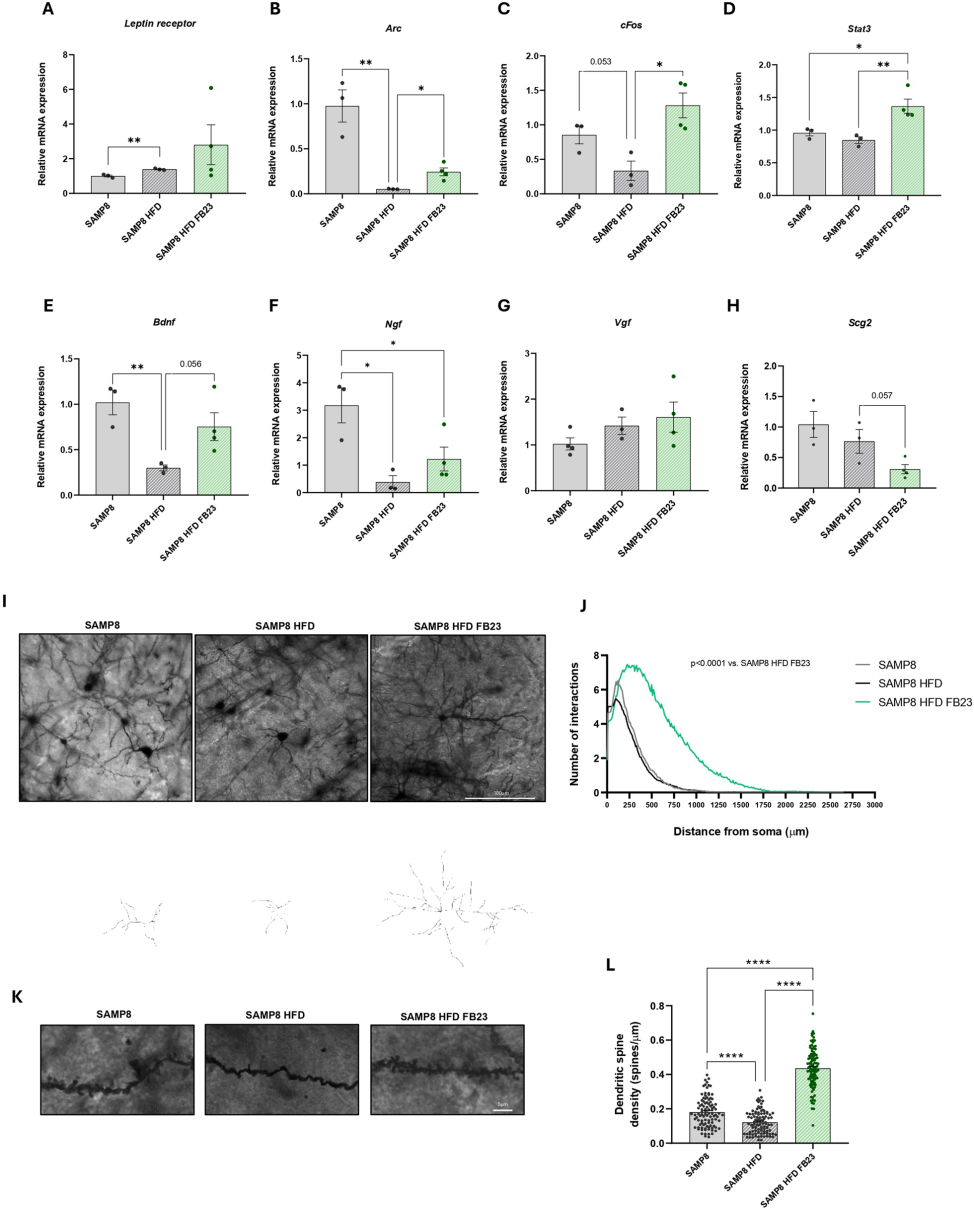
Increased synaptic plasticity in the cortical tissue of SAMP8 HFD mice after FB23 treatment. Representative gene expression in the brain of **A** *Leptin receptor*, **B** *Arc,* **C** *cFos,* **D** *Stat3,* **E** *Bdnf,* **F** *Ngf,* **G** *Vgf* and **H** *Scg2.* The relative mRNA expression levels were determined by real-time PCR. Values are expressed as mean ± SEM. Groups were compared by one-way ANOVA analysis followed using a Tukey’s post-hoc test. n = 3–4 per group; *p < 0.05; **p < 0.01. **I** Representative images and tracings of Golgi-stained cortical neurons (scale bar = 100 µm) and **J** quantification of the number of neuronal intersections vs. distance from the soma. **K** Representative images of dendritic spine density (scale bar = 5 µm) by Golgi staining and **L** quantification. Values are expressed as mean ± SEM. Groups were compared using one-way ANOVA analysis followed by Tukey’s post-hoc test. n = 100 per group for neurons and spines (**I, K**); ****p < 0.0001

Leptin signaling in the brain is involved in improved neuronal function (Kwon et al. 2016). Although the *leptin receptor* showed no differences in gene expression (Fig. 8A), a possible increased leptin sensitivity was observed in the FB23-treated group compared to the SAMP8 control groups. An increase in gene expression of immediate early genes (IEGs) involved in memory formation, such as *activity-regulated cytoskeleton-associated protein* (*Arc)* and *cellular Fos proto-oncogene* (*cFos),* and the transcription factor *signal transducer and activator of transcription 3* (*Stat3),* involved in leptin signaling, showed a significant increase in the SAMP8-HFD-FB23 group (Fig. 8B, C, D). In addition, an increase in *brain-derived neurotrophic factor* (*Bdnf)* and *nerve growth factor* (*Ngf)* neurotrophic factors, which promote synapse formation in response to learning, was observed in the FB23-treated group (Fig. 8E, F), although this was not significant for *nerve growth factor inducible* (*Vgf)* gene expression (Fig. 8G). *Secretogranin II* (*Scg2),* which is involved in the release of neuropeptides*,* showed a decreasing trend after FTO inhibition in SAMP8 HFD mice (Fig. 8H). Consistent with these results, FB23 treatment restored both neurite length and spine density in SAMP8-HFD mice compared to control groups. FB23-treated mice showed a significant increase in the number of interactions compared to the SAMP8 control and SAMP8 HFD groups, although no differences were seen between these groups (Fig. 8I, J). In terms of spine density, the SAMP8 HFD control group showed a significant decrease compared to the SAMP8 control group (Fig. 8K, L). The FB23-treated mice showed a significant improvement compared to the SAMP8 and SAMP8-HFD mice (Fig. 8K, L).

### Pharmacological FTO inhibition rescued cognitive impairment in SAMP8 HFD mice

**Fig. 9:**
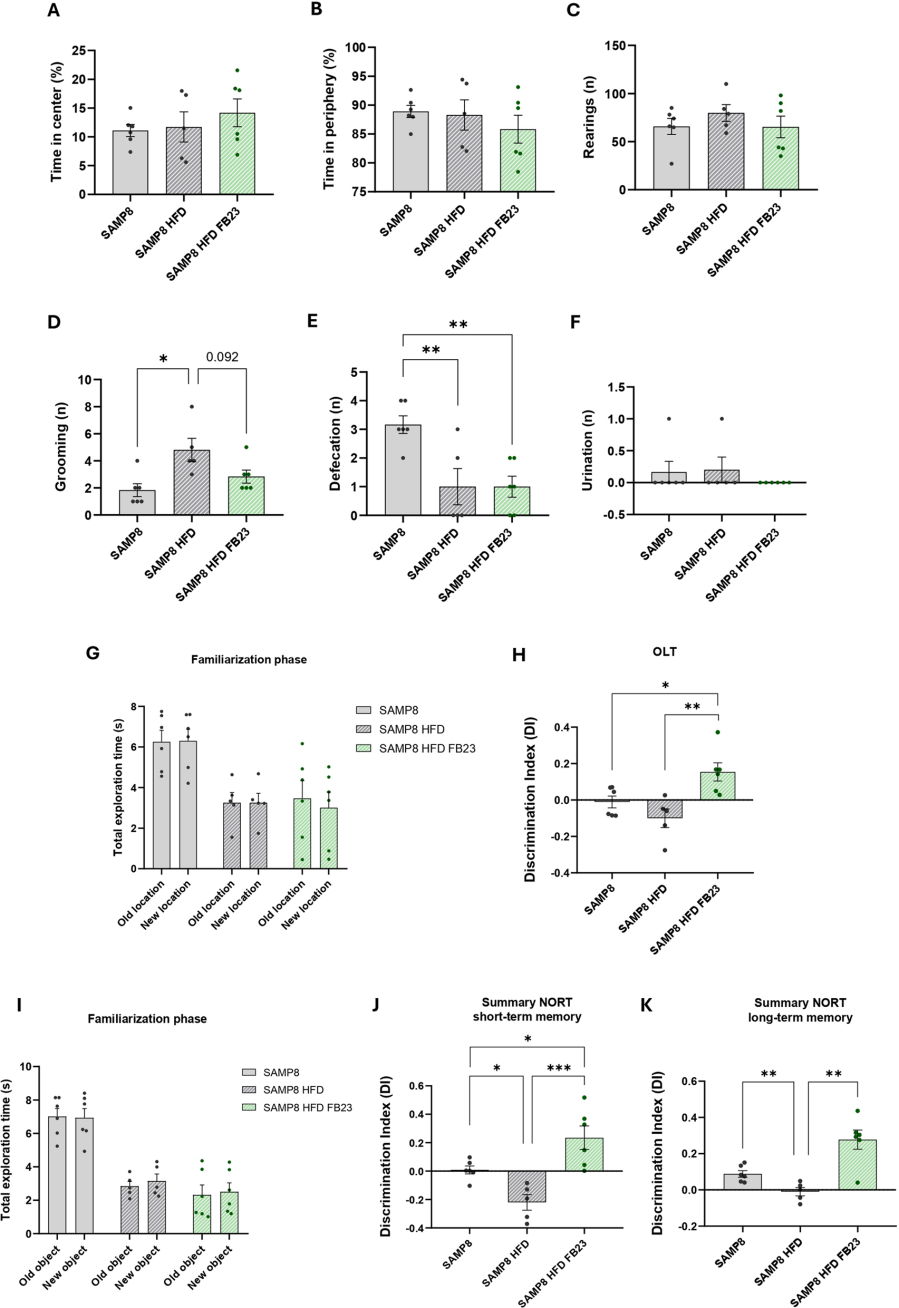
Rescued cognitive impairment in SAMP8 HFD mice after FB23 treatment. Results of the behavioral and cognitive tests and analysis. The time spent in the center (**A**) and periphery (**B**) was measured by OFT and expressed as a percentage. The number of **C** rearing, **D** grooming, **E** defecation and **F** urination were also counted. **G** Familiarization phase and **H** test of spatial memory with OLT. **I** Familiarization phase and **J** short-term memory test after 2 h and **K** long-term memory after 24 h with NORT. The discrimination index (DI) was determined for both tests. The values are given as mean ± SEM. Groups were compared using one-way ANOVA analysis followed by Tukey’s post-hoc test. n = 5–6 per group; *p < 0.05; **p < 0.01; ***p < 0.001

Several behavioral tests were performed to analyze the effect of FTO inhibition in SAMP8 HFD mice, an established model for studying cognitive decline and memory impairment (Griñán-Ferré et al. 2018). Anxiety-like behavior was first examined using the OFT. No significant differences were found in the time spent in the center and periphery of the open field (Fig. 9A, B). Although there were no changes in the number of rearings (Fig. 9C), we found that the number of grooming increased significantly in the SAMP8-HFD control, whereas it decreased after the FB23 treatment (Fig. 9D). The number of bowel movements was significantly lower in both HFD groups than in the SAMP8 control group (Fig. 9E), suggesting increased constipation due to excessive fat intake or a possible effect of the anxiety suffered during the test. In this study, urination was not affected (Fig. 9F).

Hippocampal-dependent spatial memory was investigated by performing the OLT. As no differences in exploration were observed between both locations in the familiarization phase (Fig. 9G), the animals were exposed to a relocated familiar object and the time they spent exploring the new location was measured. It was found that the treatment had a positive effect on the spatial memory of the mice compared to that of the SAMP8 and SAMP8-HFD mice (Fig. 9H). Thus, our data show that FTO inhibition ameliorated apparent cognitive impairment of SAMP8-HFD mice.

NORT was examined to analyze short-term and long-term memory. No significant differences were found in the exploration time when mice were first exposed to the objects in the training phase (Fig. 9I). SAMP8 HFD mice showed a significant decrease in DI compared to the SAMP8 group (Fig. 9J, K). An improvement in novel object recognition was observed in FB23-treated mice compared to the SAMP8 HFD group in both tests. In addition, the treated mice also showed better cognitive behavior in short-term memory compared to the SAMP8 control group (Fig. 9J, K).

## Discussion

**Fig. 10:**
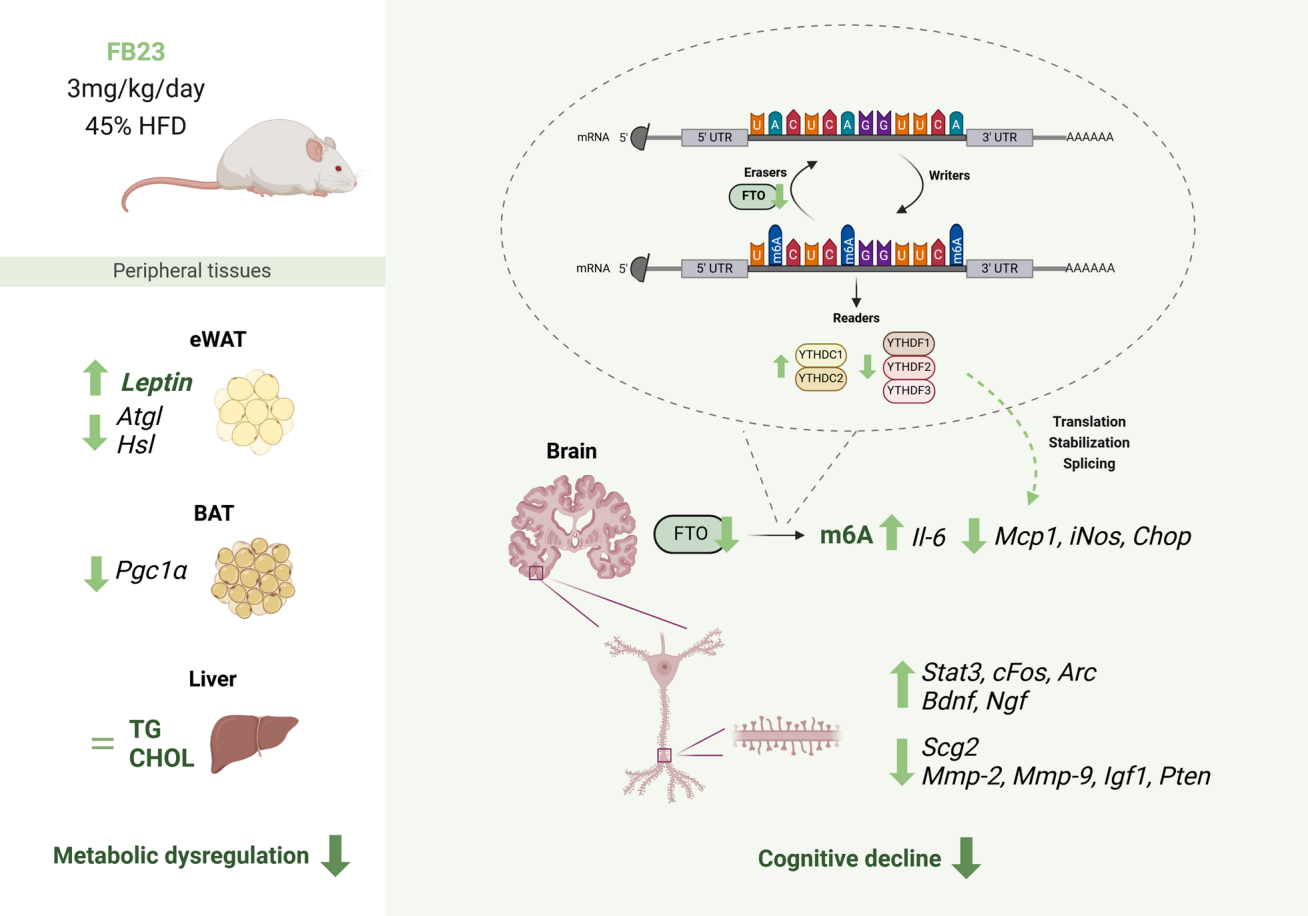
FTO inhibition ameliorates metabolic dysregulation and cognitive decline in SAMP8 mouse model after HFD

The FB23 treatment study in SAMP8-HFD-fed mice provides valuable insights into potential interventions for age-related metabolic and cognitive decline. The results demonstrated the multifaceted effects of FB23 at a 3 mg/kg dosage in an HFD-induced accelerated aging model, addressing several key aspects of metabolic dysfunction and cognitive impairment (Farooqui et al. 2012). Regarding the dose and duration of FB23 treatment, they were selected according to the published manuscript in which was found that a single dose of 3 mg/kg FB23-2 administration to Sprague Dawley rats showed a good pharmacokinetic profile with 142.5 ng/mL C_max_ and 0.4 h T_max_ values. Thus, it demonstrates an excellent amount of compound, considering its IC_50,_ which is 2.6 μM (Huang et al. 2019). Notably, the key difference between FB23-2 is a benzohydroxamic acid (-CONHOH) versus the carboxylic acid group (-COOH) presented in FB23, and this optimization was generated to improve the permeability of FB23 (Huang et al. 2019). However, in biological systems, the hydroxamic acid group of FB23-2 can undergo hydrolysis and cleave the N–O bond, converting the -CONHOH group to a -COOH. This fact resulted in the formation of FB23, effectively converting FB23-2 back to its parent compound. Also, the rate and extent of this hydrolysis may vary between species (e.g., rats vs. mice). Furthermore, the off-target effects are the same for both compounds as they can inhibit dihydroorotate dehydrogenase (hDHODH), with an IC_50_ value of 1.4 μM for FB23 and 9.2 μM for FB23-2 (Tarullo et al. 2024). We also used FB23 at 3 mg/kg because its IC_50_ is 60 nM, much better than FB23-2. Finally, very recently, the ability of FB23-2 to cross the blood–brain-barrier (BBB) in mice (Pianka et al. 2024) was demonstrated, suggesting the potential of FB23 as a therapeutic agent for CNS disorders (Fig. 10).

Glucose metabolism is critical in neurodegenerative diseases such as AD, as impaired insulin signaling and glucose utilization in the brain have been linked to cognitive decline (Amidfar et al. 2024). In this study, HFD feeding resulted in a significant increase in body weight and glucose intolerance in SAMP8 mice, as shown by the higher area under the curve (AUC) in GTT results, indicating poor glucose clearance and potential deterioration of insulin sensitivity. These results are consistent with previous studies in SAMP8 mice, showing that metabolic disorders, including glucose intolerance, typically develop at 6–9 months of age (Pačesová et al. 2022). Furthermore, treatment with FB23 did not result in a significant change in weight gain or food intake compared to the HFD control. Further GTT analysis would be required to confirm that a possible improvement in glucose tolerance without significant changes in body weight may indicate that FB23 acts via mechanisms independent of weight loss, possibly by modulating insulin signaling or glucose uptake in peripheral tissues.

Moreover, alterations in lipid metabolism and adipose tissue are increasingly recognized as factors in the development of AD (Yin 2023). Of note, treatment with FB23 further increased *leptin* levels in eWAT, suggesting possible alterations in signaling. In addition, serum leptin levels were found to be significantly elevated in the serum of HFD control mice but not in the FB23-treated group, indicating a possible increase in the sensitivity and signaling of leptin throughout the body following FTO inhibition. Dysregulation of enzymes of lipid metabolism in eWAT (*Cpt1a*, *Atgl*, *Hsl,* and *Fas*) by HFD appeared to cause increased oxidative damage (*Bip* and *Edem*) and inflammation (*Mcp1* and *Tnfα*) after FB23 treatment to alleviate tissue imbalance and regulate lipid homeostasis. In this study, BAT did not show significant changes in thermogenic and metabolic processes and, thus in oxidative damage, probably as a consequence of the age-related decrease in its function (Li et al. 2024) and the significant decrease in the main regulator of mitochondrial biogenesis *Pgc1α*. In the liver, an increasing tendency pattern of lipolysis (*Cpt1a*, *Atgl,* and *Msl*) and lipogenic enzymes (*Dgat2*, *Srebp,* and *Plin2*), as well as regulators of oxidative damage (*Catalase*, *Edem,* and *Pdi*), were observed after FTO inhibition. Furthermore, a significant increase in TG and CHOL was observed in the SAMP8-HFD groups compared to control mice, which was enhanced in FB23-treated mice. This is particularly important as SAMP8 mice have been shown to develop hepatic steatosis and alter lipid metabolism with age (Liu et al. 2008). Modulation of hepatic gene expression by FB23 could attenuate these age-related changes and contribute to improved overall metabolic health.

Epigenetic changes, including RNA methylation, play a crucial role in the development of AD (Zhang et al. 2022; Feng et al. 2024; Martinez-Feduchi et al. 2024; Xia et al. 2023; Lv et al. 2023). As for the neurological effects after FTO inhibition, a significant increase in brain m6A levels was observed after FB23 treatment, accompanied by changes in m6A-regulating enzymes. Initially, the levels of *Fto* gene were significantly decreased after treatment, which could be explained by the autoregulatory negative loop of its activity (Liu et al. 2018). Subsequently, the two m6A reader families (YTHDC and YTHDF) were analyzed. Our results showed an increase in *Ythdc1* and *Ythdc2*, whereas *Ythdf1, Ythdf2* and *Ythdf3* decreased. This suggests a complex reorganization of the m6A-dependent RNA regulation that could affect various cellular processes and disease states. Thus, as part of a compensatory mechanism, the increase could be a cellular attempt to compensate for reduced m6A demethylation caused by FTO inhibition and to promote changes in downstream biological signaling processes regulated by m6A in the brain (Kan et al. 2021; Lasman et al. 2020; Berlivet et al. 2019). Indeed, previous studies have shown that m6A methylation plays a crucial role in synaptic plasticity and memory formation (Mathoux et al. 2021). Consistent with this, our study reveals that the increased m6A levels observed upon treatment with FB23 could enhance these processes and contribute to the observed improvements in cognitive function.

Chronic neuroinflammation and oxidative stress are hallmarks of AD (Millington et al. 2014; Tönnies and Trushina 2017; Teleanu et al. 2022). In the present study, treatment with FB23 showed different effects on neuroinflammatory markers. In the FB23 treated group, there was a dramatic increase in the expression of *Il-6* compared to the SAMP8 and SAMP8-HFD controls, indicating a possible neuroprotective effect of this cytokine. While IL-6 is often associated with inflammation, it also has several beneficial effects as an anti-inflammatory chemokine (Steensberg et al. 2003; Gleeson et al. 2011). It plays a crucial role in metabolic regulation, promoting lipolysis, glucose uptake, and insulin sensitivity (Febbraio et al. 2004; Ellingsgaard et al. 2011). IL-6 mediates many anti-inflammatory and insulin-sensitizing effects of physical exercise, contributes to tissue repair and regeneration, and supports immune system function (Wedell-Neergaard et al. 2019; Pedersen and Febbraio 2012, 2008; Pedersen et al. 2004). These findings highlight the intricate relationships between diet, genetic factors, and molecular pathways in the context of metabolic disorders and neurodegenerative diseases. Similarly, IL-6 has been shown to be a key component in neuronal development and survival by activating STAT3-mediated stem cell formation, and it is considered a neurotrophic factor when acting in concert with NGF in neuronal outgrowth (Kummer et al. 2021). Furthermore, the reduction in *Mcp1 and iNos* after FTO inhibition is remarkable. High levels of these two neuroinflammatory markers have been described as age promoting immune factors that impair memory function (Bettcher et al. 2019; Justo and Suemoto 2022). While there were no significant changes in oxidative stress markers such as *Bip* or *Catalase*, changes in ER stress occurred after FB23 treatment due to the reduction of *Chop*, which is thought to be involved in neuronal apoptosis pathways (Xu et al. 2021). These findings are particularly relevant in the context of SAMP8 mice, in which increased neuroinflammation and oxidative stress were observed as early as 3 months of age (Pačesová et al. 2022). Interestingly, a study by Hess et al. showed that FTO deficiency reduced neuroinflammation in a mouse model of multiple sclerosis (Lv et al. 2018). Our results suggest that FTO inhibition may have a broader anti-inflammatory effect on the CNS. If so, reducing inflammatory markers could slow down the progression of age-related neurodegeneration in this model.

Synaptic dysfunction is considered an early event in AD and is strongly correlated with cognitive decline (Pelucchi et al. 2022). Here, one of the most striking findings is the significant increase in dendritic spine density and number of interactions observed in the FB23-treated group. This indicates improved enhanced synaptic plasticity and potentially improved cognitive function. FTO inhibition resulted in decreased expression of *Mmps*, which has shown promise as a therapeutic strategy for neuroprotection (Chen et al. 2009; Chaturvedi and Kaczmarek 2014), and potential changes in IGF1 signaling (Kok and Barton 2021), highlighting the significant decrease in *Pten*, the inhibition of which has been studied to protect against cognitive impairment (Tan et al. 2017), and a reduction of downstream signaling pathway of AKT and NF-κB target genes, which has been shown to be protective against HFD-induced metabolic dysregulation (Tseng 2024; Entezari et al. 2022). Significantly, FB23 treatment increased the expression of IEGs involved in memory formation, such as *Arc* and *cFos,* as well as the transcription factor *Stat3,* suggesting a general activation of leptin signaling via its receptor in the brain. The expression of genes related to synaptic plasticity, such as the neurotrophic factors *Bdnf* and *Ngf*, was also significantly increased after treatment. *Scg2* in particular, was significantly reduced, further supporting this hypothesis (Lim et al. 2021). These results are particularly significant, as SAMP8 mice typically exhibit reduced neurogenesis and synaptic plasticity as early as 3 months of age (Pačesová et al. 2022). The ability of FB23 to improve synaptic plasticity and increase the expression of neurotrophic factors could counteract these age-related changes and contribute to improved cognitive function.

Interestingly, another significant finding was the rescue of cognitive impairment in SAMP8 HFD mice by treatment with FB23. The treated mice showed improved performance on both short- and long-term and spatial memory tasks. This suggests that inhibiting FTO may have neuroprotective effects in the context of HFD-induced cognitive decline. This is consistent with work by Walters et al., who found that FTO regulates dopaminergic signaling and synaptic plasticity in the midbrain (Walters et al. 2017). Not only by improving neuroplasticity, but also by alleviating neuroinflammation, FTO has been studied to positively correlate with working memory and anxiety-like behavior (Kummer et al. 2021). Indeed, our study showed that treatment with FB23 significantly reduced the level of self-grooming performed by HFD mice, an indicator of stress and anxiety. We also found a significant decrease in defecation in the HFD groups compared to the SAMP8 control group in the OFT. Considering there were no differences in food intake between the groups, this could be due to constipation induced by excessive fat intake via the reduction of colonic mucus in mice (Mukai et al. 2020) or as an indicator of fear and anxiety suffered during the test.

Furthermore, improving synaptic plasticity could have important implications for treating cognitive decline in aging and neurodegenerative diseases. The observed increase in m6A levels in the brain following treatment with FB23 is also particularly intriguing. Recent research has emphasized the importance of m6A methylation in synaptic plasticity and memory formation. Widagdo et al. have shown that dynamic regulation of m6A is critical for memory consolidation in mice (Widagdo et al. 2016). Our findings of enhanced synaptic plasticity and cognitive performance by FB23 treatment are consistent with this new understanding of the role of m6A's in cognition. These results are particularly noteworthy as SAMP8 mice typically show significant cognitive decline at 6 months of age (Pačesová et al. 2022). The ability of FB23 to improve cognitive performance is particularly noteworthy given the accelerated cognitive decline that characterizes the SAMP8 model. These results suggest that FB23 may target key mechanisms involved in the cognitive decline associated with AD, possibly through its effect on synaptic function and neurotrophic factor expression.

## Implications and future directions

The intricate relationship between metabolic stress, epigenetic regulation, and cognitive function has been highlighted by recent research on the FTO gene. Interestingly, the inhibition of FTO appears to ameliorate cognitive deficits without significantly affecting body weight. This suggests that m6A-dependent processes in the brain may be a promising way to improve cognitive performance in metabolic disorders. This finding opens up several exciting research opportunities. First, there is an urgent need to elucidate the specific molecular mechanisms by which FTO inhibition improves synaptic plasticity and cognitive performance. Understanding these mechanisms could provide valuable insights into learning and memory's fundamental processes. Second, the long-term effects of FTO inhibition on metabolic and cognitive parameters need to be thoroughly investigated***.*** While short-term studies show promising results, evaluating the sustainability and safety of this approach over more extended periods is essential. Finally, the potential of FTO inhibitors as therapeutic agents for age-related cognitive decline and metabolic disorders is a tantalising prospect that should be explored further. This could lead to the development of novel interventions that simultaneously address cognitive impairment and metabolic dysfunction, two increasingly prevalent health problems in our aging population. By pursuing these avenues of research, we may discover new strategies to improve cognitive function in individuals with metabolic disorders, improving the quality of life for millions of people worldwide.

## Supplementary Information

Supplementary material 1.

## Data Availability

No datasets were generated or analysed during the current study.
