# Supplementary material for: FTO inhibition mitigates high-fat diet-induced metabolic disturbances and cognitive decline in SAMP8 mice
Source: Mol Med. 2025 Feb 21;31:73. doi: 10.1186/s10020-025-01126-4 (PMC11843768; doi:10.1186/s10020-025-01126-4)
Supplement: Supplementary file 1 — Supplementary material 1. [file 10020_2025_1126_MOESM1_ESM.docx]

**Table S1.** Formulation of the conventional diet used in this study (Inotiv, Madison, WI, USA, #2018 Teklad Global 18% Protein Rodent Diets).

| Macronutrients* | Amount | Unit |
| --- | --- | --- |
| Crude protein | 18.4 | % |
| Fat (ether extract) ^a^ | 6.0 | % |
| Carbohydrates (available) ^b^ | 44.2 | % |
| Crude fiber | 3.8 | % |
| Neutral detergent fiber ^c^ | 14.7 | % |
| Ash | 5.5 | % |
| Energy density ^d^ | 3.1 (13.0) | kcal/g (kJ/g) |
| Calories from protein | 24 | % |
| Calories from fat | 18 | % |
| Calories from carbohydrate | 58 | % |

^a^ Ether extract is used to measure fat in pelleted diets, while an acid hydrolysis method is required to recover fat in extruded diets. Compared to ether extract, the fat value for acid hydrolysis will be approximately 1% point higher.

^b^ Carbohydrate (available) is calculated by subtracting neutral detergent fiber from total carbohydrates.

^c^ Neutral detergent fiber is an estimate of insoluble fiber, including cellulose, hemicellulose, and lignin. Crude fiber methodology underestimates total fiber.

^d^ Energy density is a calculated estimate of metabolizable energy based on the Atwater factors assigning 4 kcal/g to protein, 9 kcal/g to fat, and 4 kcal/g to available carbohydrate.

*Minerals, amino acids, vitamins, fatty acids and other ingredients such as cholesterol are not detailed in this table, refer to 2018 Teklad Global 18% Protein Rodent Diets.

**Table S2.** Formulation of the HFD used in this study (Research Diets Inc., NJ, USA, #D12451).

| **Class description** | **Ingredients** | **Grams** |
| --- | --- | --- |
| Protein | Casein, Lactic, 30 Mesh | 200.00 g |
| Protein | Cystine, L | 3.00 g |
| Carbohydrate | Sucrose, Fine Granulated | 176.80 g |
| Carbohydrate | Lodex 10 | 100.00 g |
| Carbohydrate | Starch, Corn | 72.80 g |
| Fiber | Solka Floc, FCC200 | 50.00 g |
| Fat | Lard | 177.50 g |
| Fat | Soybean Oil, USP | 25.00 g |
| Mineral | S10026B | 50.00 g |
| Vitamin | Choline Bitartrate | 2.00 g |
| Vitamin | V10001C | 1.00 g |
| Dye | Dye, Red FD&C #40, Alum. Lake 35-42% | 0.05 g |
|  | **Total:** | 858.15 g |
